# Machine learning classification of ADHD and HC by multimodal serotonergic data

**DOI:** 10.1038/s41398-020-0781-2

**Published:** 2020-04-07

**Authors:** A. Kautzky, T. Vanicek, C. Philippe, G. S. Kranz, W. Wadsak, M. Mitterhauser, A. Hartmann, A. Hahn, M. Hacker, D. Rujescu, S. Kasper, R. Lanzenberger

**Affiliations:** 1 Department of Psychiatry and Psychotherapy, Medical University of Vienna, Vienna, Austria; 2 Department of Biomedical Imaging and Image-guided Therapy, Division of Nuclear Medicine, Medical University of Vienna, Vienna, Austria; 3 Department of Rehabilitation Sciences, Hong Kong Polytechnic University, Hung Hom, Hong Kong; 4 Center for Biomarker Research in Medicine CBmed, Graz, Austria; 5 Ludwig Boltzmann Institute Applied Diagnostics, Vienna, Austria; 6 Department of Psychiatry, University of Halle, Halle, Germany

**Keywords:** Molecular neuroscience, ADHD, Clinical genetics

## Abstract

Serotonin neurotransmission may impact the etiology and pathology of attention-deficit and hyperactivity disorder (ADHD), partly mediated through single nucleotide polymorphisms (SNPs). We propose a multivariate, genetic and positron emission tomography (PET) imaging classification model for ADHD and healthy controls (HC). Sixteen patients with ADHD and 22 HC were scanned by PET to measure serotonin transporter (SERT‘) binding potential with [^11^C]DASB. All subjects were genotyped for thirty SNPs within the *HTR1A, HTR1B, HTR2A* and *TPH2* genes. Cortical and subcortical regions of interest (ROI) were defined and random forest (RF) machine learning was used for feature selection and classification in a five-fold cross-validation model with ten repeats. Variable selection highlighted the ROI posterior cingulate gyrus, cuneus, precuneus, pre-, para- and postcentral gyri as well as the SNPs *HTR2A* rs1328684 and rs6311 and *HTR1B* rs130058 as most discriminative between ADHD and HC status. The mean accuracy for the validation sets across repeats was 0.82 (±0.09) with balanced sensitivity and specificity of 0.75 and 0.86, respectively. With a prediction accuracy above 0.8, the findings underlying the proposed model advocate the relevance of the SERT as well as the *HTR1B* and *HTR2A* genes in ADHD and hint towards disease-specific effects. Regarding the high rates of comorbidities and difficult differential diagnosis especially for ADHD, a reliable computer-aided diagnostic tool for disorders anchored in the serotonergic system will support clinical decisions.

## Introduction

The most common neurodevelopmental disorder, attention-deficit and hyperactivity disorder (ADHD), affects up to 10% of children with symptoms often persisting throughout the whole lifespan and predisposes to comorbidities like major depressive disorder (MDD)^1^. However, substantial fluctuation of prevalence was reported between and across nations, likely owed to disputed diagnostic criteria that are mostly based on behavioral symptoms rather than objective biomarkers. While pathognomonic for many psychiatric disorders, the lack of biomarkers for ADHD is particularly baneful due to the overlap of core symptoms with other frequent psychiatric disorders as mood, anxiety and personality disorders. Diagnosis of adult ADHD is further hindered by retrospective assessment of symptoms in the childhood. Disputes among opinion leaders on ADHD and debates over misuse of ADHD treatment like methylphenidate (MPH) have encouraged research exploring objective over subjective ADHD predictors, so far with modest success^2,3^.

Genetics were expected to resolve disparate findings and explain heterogeneity, especially in ADHD with heritability estimated to exceed 70%^4,5^. Some candidate gene studies associated variants implicated in the monoaminergic neurotransmission with ADHD^6,7^, while the GWAS mostly highlighted genes that previously received less attention and are trickier to mesh with established etiologic theories^8^. However, genetic studies did not impact ADHD diagnosis or treatment yet^9^. Consequently, the translation to the clinic is lacking so far.

Neuroimaging and data-driven diagnostics that naturally come along with it were considered a corrective to the issues of subjective symptoms and heterogeneity. Basic tools like electro-encephalography (EEG) as well as more advanced techniques as magnetic resonance imaging (MRI) have now been fully established in ADHD research^10^. While misbalance of dopaminergic and noradrenergic neurotransmission is putatively the main biological substrate of ADHD, data on in vivo neuroreceptor binding are scarce due to the resource intensive nature of positron emission tomography (PET). While serotonergic neurotransmission is considered a pivotal substrate of affective disorders, the role of serotonin is not sufficiently understood in ADHD^11,12^. Data from animal models as well as pharmacological and genetic studies point toward involvement of serotonin in ADHD and atomoxetine, a well-established drug in ADHD treatment, has been demonstrated to block the serotonin transporter in addition to its noradrenergic properties^13^. Emotional dysregulation with mood swings and irritability, closely linked to serotonergic pathways, has lately been discussed as an additional core symptom of ADHD^14,15^. Additionally, comorbid mood disorders are frequent in ADHD. Nevertheless, only few PET studies have targeted the serotonin system in ADHD so far. Earlier studies on the serotine transporter (SERT) binding did not support differences while recently altered interregional connectivity of SERT binding in the hippocampus and precuneus of ADHD patients compared to control subjects was demonstrated^16^.

With the influx of advanced statistics into neuropsychiatric research, a copious amount of machine learning studies targeted ADHD classification. Algorithms based on EEG and MRI features reported accuracies ranging from hardly above chance level to beyond 90%^10^. However, no studies combined imaging and genetic predictors up to this point. A reliable diagnostic tool for ADHD may be especially relevant to precision medicine in psychiatry. Since serotonergic transmission has to some extent been demonstrated to pilot the disorders, the focus of this study was classification of ADHD and healthy individuals based on multimodal serotonergic data.

## Methods

### Subjects

ADHD subjects derive from a previously reported study on SERT binding measured with [^11^C]DASB^16^.

In short, 16 patients with adult ADHD (aged 31.9 ± 10.9 standard deviation (SD), seven females) were recruited through the outpatient clinic for ADHD and affective disorders at the Department of Psychiatry and Psychotherapy, Medical University of Vienna. Twenty-two healthy control subjects (aged 33.19 ± 10.3 SD, nine females) were recruited through advertisement at the Department of Psychiatry and Psychotherapy. ADHD patients were required to be free of neuropsychiatric medication for at least three months. None of the HC were previously exposed to any psychopharmacologic treatment. All study related procedures were approved by the Ethics Committee of the Medical University of Vienna. All participants consented in written form to partake in the study after extensive explanation of the study protocol.

Subjects were screened for any somatic or neurological disorder by assessment of physical and neurological status, laboratory tests including urine drug and pregnancy tests and electrocardiography. Comorbid psychiatric disorders were assessed with the structured clinical interview for DSM-IV (SCID-I, SCID-II). Subjects with severe comorbidities or any substance abuse or addiction other than nicotine were excluded. ADHD symptomatology was evaluated by Conners’ Adult ADHD Diagnostic Interview (CAADID, Conners 1999).

### Genotyping procedures

Genotyping protocols were published previously, please see for details ref.^17^. In summary, EDTA blood tubes of 9 ml were collected and the QiaAmp DNA blood maxi kit was applied for DNA isolation (Qiagen, Hilden, Germany). The iPLEX assay was used for genotyping on a mass spectrometer (MassARRAY MALDI-TOF). Typer 3.4 (Sequenom, San Diego, CA, USA) was utilized for genotype assignment after selection of the allele-spcific extension products. Quality control required to surpass a threshold of 80% individual and 99% SNP call rate identity of genotyped CEU trios (Coriell Institute for Medical research, Camden, NJ).

Thirty SNPs of four genetic key players of the serotonergic system, the *HTR1A*, *HTR1B*, *HTR2A* and *TPH2* genes, were selected for this analysis based on the literature. All SNPs were coded numerically for the number of minor alleles, therfore ranging from 0 to 2. SNPs were determined based on the literature. For an overview of baseline characteristics, including genotypes (Table 1).

**Table 1:** Baseline characteristics with sex, age and genotypes for the total sample, HC and ADHD.

*n*	Total, 38	HC, 22	ADHD, 16
Age (mean ± SD)	32.51 ± 10.5	33.19 ± 10.3	31.9 ± 10.9
Sex (female/male)	16/22	9/13	7/9
Mean Global BP_ND_	0.37 ± 0.36	0.37 ± 0.36	0.36 ± 0.35
*HTR1A* rs6295	13/16/9	6/10/6	3/6/7
*HTR1A* rs878567	13/16/9	6/10/6	7/6/3
*HTR1A* rs1423691	13/16/9	6/10/6	7/6/3
*HTR1A* rs10042486	13/16/9	6/10/6	3/6/7
*HTR1B* rs6296	4/18/16	2/10/10	2/8/6
*HTR1B* rs6298	16/18/4	10/10/2	6/8/2
*HTR1B* rs130058	21/15/2	16/4/2	5/11/0
*HTR1B* rs13212041	25/12/1	13/8/1	12/4/0
*HTR2A* rs6311	11/18/9	7/13/2	4/5/7
*HTR2A* rs6313	11/18/9	7/13/2	4/5/7
*HTR2A* rs1328684	16/20/2	4/16/2	12/4/0
*HTR2A* rs1923886	10/19/9	5/10/7	5/9/2
*HTR2A* rs2224721	17/16/5	8/10/4	9/6/1
*HTR2A* rs2770296	23/13/2	12/9/1	11/4/1
*HTR2A* rs6561332	9/20/9	4/11/7	5/9/2
*HTR2A* rs6561333	13/18/2	10/6/1	3/12/1
*HTR2A* rs7322347	11/22/5	7/12/3	4/10/2
*HTR2A* rs7984966	21/17/0	13/9/0	8/8/0
*HTR2A* rs7997012	13/19/6	7/11/4	6/8/2
*HTR2A* rs9316233	18/13/7	8/9/5	10/4/2
*HTR2A* rs9534495	9/18/11	5/10/7	4/8/4
*TPH2* rs1386493	28/8/2	16/5/1	12/3/1
*TPH2* rs1386497	29/7/2	16/5/1	13/2/1
*TPH2* rs1487275	22/12/4	11/8/3	11/4/1
*TPH2* rs1487278	26/10/2	16/4/2	10/6/0
*TPH2* rs1843809	29/7/2	15/6/1	14/1/1
*TPH2* rs4570625	25/12/1	17/5/0	8/7/1
*TPH2* rs7305115	13/19/6	9/9/4	4/10/2
*TPH2* rs10879352	17/14/7	10/7/5	7/7/2

For each single nucleotide polymorphism counts for major allele homozygotes, heterozygotes and minor allele homozygotes are provided (in this order).

*ADHD* attention-deficit and hyperactivity disorder, *MDD* major depressive disorder, *HC* healthy control, *SD* standard deviation, *BP*_*ND*_ non-displaceable binding potential.

### PET data acquisition

All PET and MRI scans were carried out at the Department of Biomedical Imaging and Image-guided Therapy, Division of Nuclear Medicine, Medical University of Vienna. A full-ring scanner (General Electric Medical Systems, Milwaukee, WI, USA) in 3D acquisition mode was used. For all subjects, the state-of-the-art radiotracer [11 C]DASB was used to quantify SERT binding as protocolled previously^18^. In summary, for tissue attenuation correction a transmission scan was obtained for five minutes with retractable 68 Ge rod sources. Data acquisition of the actual scan started with a bolus i.v.-injection of [11 C]DASB. A series of 50 consecutive time frames (12 × 5 s, 6 × 10 s, 3 × 20 s, 6 × 30 s, 4 × 1 min, 5 × 2 min, 14 × 5 min) was carried out, resulting in a measurement time of 90 min in total. FORE-ITER, an iterative filtered back-projection algorithm, was used for reconstructing the measured data in volumes of 35 transaxial sections (128 × 128 matrix). For this step, the spatial resolution was at 4.36 mm full-width at half maximum 1 cm next to the center of the field of view.

### SERT quantification

The protocol for data quantification was reported previously, including preprocessing carried out in SPM12 (Wellcome Trust Centre for Neuroimaging, London, UK; http://www.fil.ion.ucl.ac.uk/spm/)^16^. In summary, the means of all time frames without visually observable head motion was used for realignment of each time frame of the dynamic PET scans. All subjects also underwent MRI scans on a 3 Tesla Philips scanner (Achieva, 3D T1 FFE weighted sequence, 0.88 mm slice thickness, 0.8 × 0.8 mm in-plane resolution). Summed PET images (integral) from realigned data was co-registered to T1-weighted images. Next, spatial normalization of the T1-weighted images was performed. Transformation of the co-registered PET images into MNI standard space was achieved by application of the obtained transformation matrices to the dynamic PET data. Finally, computation of voxel-vise images of BP_ND_ values was carried out with PMOD image analysis software, version 3.509 (PMOD Technologies Ltd., Zurich, Switzerland; http://www.pmod.com) and the multilinear reference tissue model with two parameters (MRTM2)^19^. The cerebellar grey matter without vermis and venous sinus was assigned as the reference region due to negligible availability of SERT in this region^20,21^.

Non-displaceable binding potential (BP_ND_) values were extracted for regions defined according to the automated anatomical atlas (AAL). Mean values were calculated from BP_ND_ for the left and right hemispheres. Thus, a total of 49 cortical and subcortical ROI was included in the analyses.

### Statistics

A classification model for ADHD and HC was computed with genetic predictors, imaging predictors, all predictors as well as the top performing predictors, for each fold, respectively.

Computations were performed with the statistical software “R” (https://www.R-project.org/). The package “randomForest” was used for application of the eponymous algorithm (RF)^22,23^. In short, RF is an ensemble tree classification tool that randomly selects subsamples of observations and builds a decision tree for optimal splitting of these observations according to an outcome variable by a combination of predictors. For each split, the best performing predictor out of a random selection is applied. Generally, a higher number of predictors allowed for selection leads to optimal splits but also low diversity of the individual trees. Therefore, restricting the number of features can generate models that perform worse in the training set but are more flexible when exposed to new data. Here, 3000 trees were grown (ntree = 3000) for each model to enable multiple predictions for all patients. Classification was performed with a five-fold cross-validation (CV) design to allow optimal validation in absence of an independent test set^24^. If hyperparameters must be tuned, nested CV is the gold-standard technique to prevent data leakage from training to the validation phase. For RF only the number of features randomly selected at each split (mtry) can be optimized; however, there is a standard of using the square root of the number of predictors. To prevent overfitting, no optimization of mtry was performed for this analysis.

For variable selection, a combination of established algorithms “Boruta” and “varSelRF” for “R” were used^23,25^. Comparable to permutation-based importance evaluations, “Boruta” doubles the predictors included in the model by generating “shadow predictors” that show randomly interchanged values for each observation. Then 500 iterations of RF are run and only those predictors performing better than the best “shadow predictor” by a *p*-value threshold of 0.01 are preserved. These relevant predictors were then included in a backwards variable elimination algorithm, “varSelRF”. The best performing combination of predictors was then applied to the test set corresponding to each fold of the CV.

**Fig. 1:**
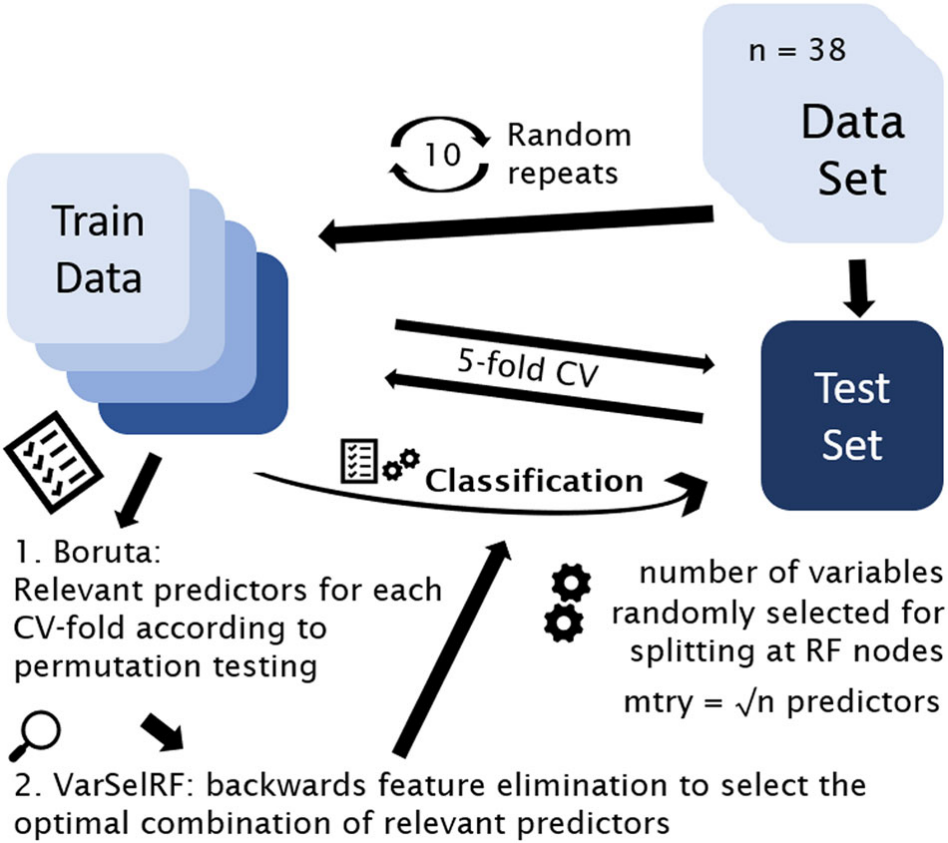
Graphical representation of the five-fold CV design. CV was performed with standard settings for variables randomly selected for splitting at each node (mtr\y = square root of the number of predictors) and variable selection based on imputation testing as provided by “Boruta” and backwards feature elimination as provided by “VarSelRF”. The top perfroming predictors of each training set were used for classification of the respective test set. The whole CV procedure was repeated ten times and results were averaged. CV cross-validation.

The whole CV procedure was repeated ten times and average accuracy is reported. See also Fig. 1 for a synopsis of the CV design.

There is no established method of power calculation for RF. Research indicated stable predictive capabilities of RF and comparable machine learning algorithms when enough observations are available, even in high dimensional data with the number of variables surpassing that of observations^26,27^. For this dataset, a ratio of 79 predictors to 38 subjects was observed.

In addition to the results produced by RF, a mixed model was computed with the “lme” package for “R”. Linear mixed regression models for BP_ND_ with diagnosis, ROI and the most informative genetic predictors included as fixed effects and subject as random effect were built. Main and interaction effects (up to three-way) were computed. Mixed model results were corrected for the number of tests and models with a corrected threshold of *p* ≤ 0.001. Based on these results, logistic regression models for each ROI and SNP with diagnosis as outcome variable and the respective ROI/SNP interaction term were computed. Logistic regression results were not corrected.

## Results

### Classification

Using all available predictors in a five-fold CV model yielded an accuracy of 0.80 (±0.13). Restricting the predictors to either ROI or SNPs only, accuracies dropped to 0.58 (±0.15) and 0.62 (±0.15), respectively. Using only the top performing predictors boosted the accuracy to 0.76 (±0.13) and 0.71 (±0.16) for SNPs and ROI, respectively. Optimal results were achieved combining the selected SNPs and ROI, with an accuracy of 0.82 (±0.09) and positive and negative predictive values (PPV and NPV) of 0.80 and 0.83, indicating the probability of correct classification for and ADHD and HC, respectively

For a detailed overview of the classification outcome with all evaluation parameters (Table 2).

**Table 2:** Performance evaluators of the classification model for ADHD and HC.

Classification HC vs ADHD	Sens	Spec	PPV	NPV	Accuracy
SNPs (*n* = 30)	0.56	0.66	0.55	0.67	0.62 (±0.15)
ROI (*n* = 49)	0.50	0.73	0.50	0.61	0.58 (±0.15)
All (*n* = 79)	0.82	0.80	0.75	0.86	0.80 (±0.13)
FS SNPs	0.71	0.80	0.72	0.79	0.76 (±0.13)
FS ROI	0.65	0.76	0.66	0.75	0.71 (±0.16)
FS SNP + ROI	0.75	0.86	0.80	0.83	0.82 (±0.09)

Sensitivity, specificity, PPV, NPV and accuracy are provided for models based on only ROI or SNP predictors, all predictors, and predictors highlighted by feature selection. All results are averaged over 10 repeats of five-fold cross-validation.

*ADHD* attention-deficit and hyperactivity disorder, *HC* healthy control, *PPV* positive predictive value, *NPV* negative predictive value, *FS* feature selection.

### Variable selection

**Fig. 2:**
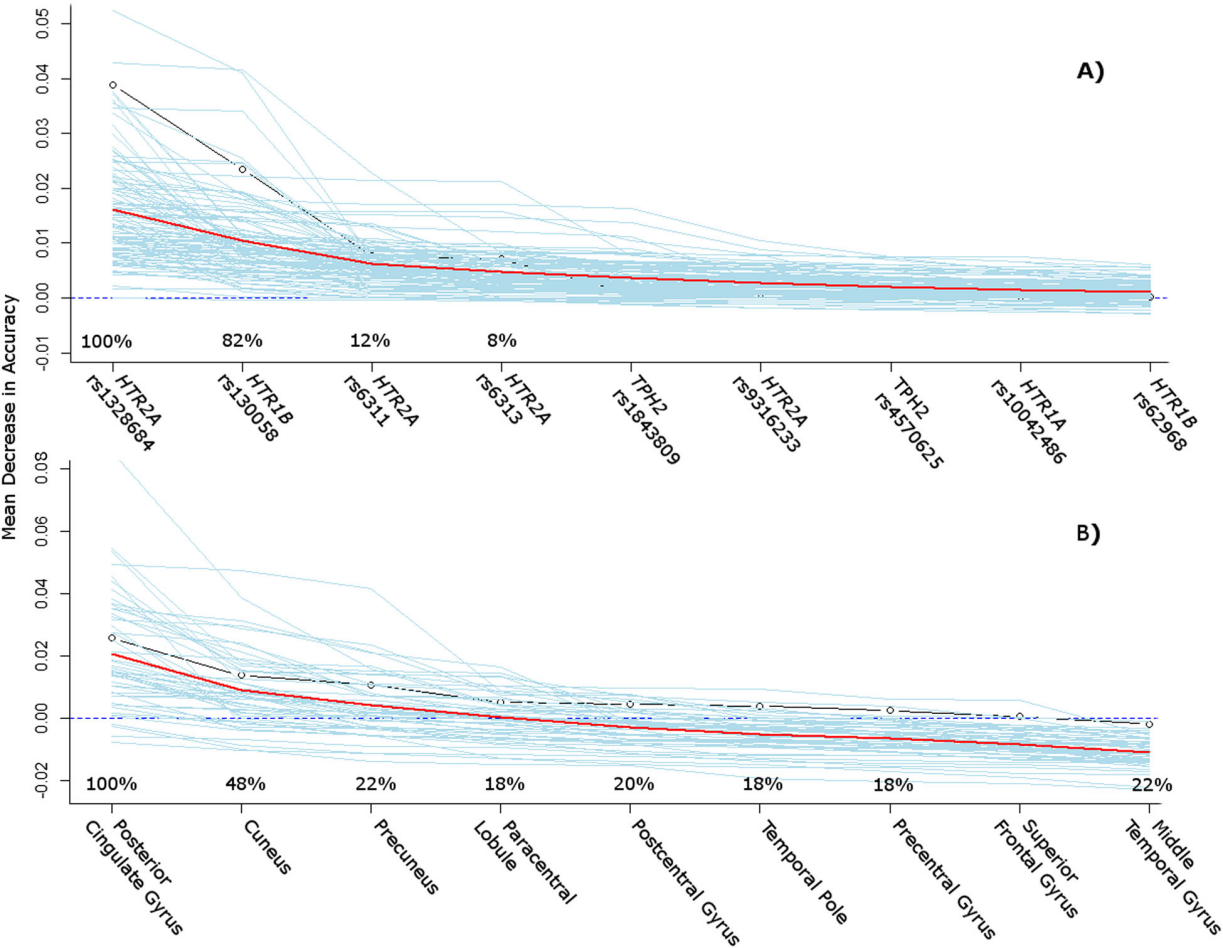
Variable importance measurement from RF by MDA for the total dataset (*n* = 38) with 5000 trees grown and 100 random permutations. Predictors are ordered by declining information criterion for classification accuracy. Blue lines each represent drops in MDA after a random permutation of the predictor values and the red line shows averages of all 100 permutations. The precentages indicate how often the top predictors for the whole dataset were selected in the training sets of the cross-validation runs and thus were used for classification of the test sets. **a** shows results for SNPs while **b** shows ROI predictors. RF RandomForest, MDA mean decrease in accuracy, SNP single nucleotide polymorphisms, ROI region of interest.

Concerning variable selection, most predictors showed a high agreement between training sets. The posterior cingulate gyrus was selected by 100% of models, followed by cuneus (48%), precuneus (22%), middle temporal gyrus (22%), precentral gyrus (20%), paracentral lobule (18%), postcentral gyrus (18%) and temporal pole (18%). Among the SNPs, *HTR2A* rs1328684 was selected by all models, followed by *HTR1B* rs130058 (82%). Other than that, only rs6311 (12%) and rs6313 (8%) were selected, which showed identical allelic expression. For a graphical depiction of importance measurements and probability rates of the top scoring predictors being selected in the training sets of each fold (Fig. 2).

### Mixed model and logistic regression statistics

Mixed model revealed three-way associations for *HTR1B* SNP rs130058 (F = 1.67, *p* < 0.0001), *HTR2A* SNP rs1328684 (F = 1.61, *p* = 0.001), *HTR2A* rs6313 (F = 2.38, *p* < 0.0001) and *TPH2* rs1843809 (F = 2.08, *p* < 0.0001). Significant results of the mixed models are presented in Table 3, section A.

**Table 3:** A) Linear mixed model results for effects of diagnosis, ROI and the top performing SNPs on BP_ND_. B) Binomial logistic regression results for classification of diagnosis.

A) Mixed model	**DF** numerator	**DF** denominator	F value	*p*-value
Group ∗ ROI ∗ *HTR2A* rs1328684	49	1666	1.61	0.001
Group ∗ ROI ∗ *HTR1B* rs130058	49	1666	9.49	<0.0001
Group ∗ ROI ∗ *HTR2A* rs6313	49	1666	2.38	<0.0001
Group ∗ ROI ∗ *TPH2* rs1843809	49	1666	2.08	<0.0001

B) Logistic regression model	Estimator	Std. Error	Z value	*p*-value
Temporal Pole ∗ *HTR1B* rs130058	−21.76	9.52	−2.29	0.022
PCG ∗ *HTR1B* rs130058	−17.93	8.38	−2.14	0.032

Models were built for each of the top performing ROI and, respectively, for each interaction with performing SNPs, according to the machine learning classification model. Interaction effects are marked with ∗. Only significant results are shown (uncorrected). Mixed model results with *p* ≤ 0.001 stayed significant after correction for multiple testing. None of the logistic regression results withstood correction.

*ROI* region of interest, *SNP* single nucleotide polymorphisms, *BP*_*ND*_ non-displaceable binding potential, *DF* Degrees of Freedom, *PCG* Posterior Cingulate Gyrus.

Logistic regression revealed interaction effects for the temporal pole (z = −2.29, *p* = 0.022) as well as posterior cingulate gyrus (z = −2.14, *p* = 0.032) with *HTR1B* rs130058, none of which remained significant after correction. Significant results of the logistic regression models are presented in Table 3, section B.

## Discussion

Evaluating PET imaging and genetic predictors anchored within the serotonergic system, a moderate accuracy of 0.82 could be achieved for classification of ADHD and HC. Beyond the utility as a diagnostic tool, these results advocate different and recognizable serotonergic properties for ADHD and HC.

Predictor evaluation based on importance can adumbrate interaction effects that would not reach statistical significance in conventional association as thousands of predictor combinations are assessed for model building. Concerning the most prominent features in this analysis, SERT BP_ND_ within the three ROI posterior cingulate gyrus, cuneus and precuneus as well as SNPs rs130058 of *HTR1B* and rs1328684 of *HTR2A* were selected consistently by variable importance measures. All the anatomical structures labelled by these ROIs have previously been implicated in ADHD pathology as part of the default mode network (DMN)^28^. Altered DMN activation during sustained attention paradigms in ADHD could thereby be partly redeemed by methylphenidate^29–31^. Also both SNPs rs130058 and rs1328684 were implicated to mediate DMN abnormalities measured by MRI in a recent candidate gene study in PTSD^32^. *HTR1B* rs130058 was previously associated with ADHD as well as frequent comorbidities as substance dependence disorders^33,34^. Although *HTR1B* was highlighted as a top gene by candidate gene reviews in ADHD^35^, findings for rs130058 were overall inconsistent^36–38^. In this sample, ADHD patients showed an increased frequency of rs1328684 minor alleles (1.69 vs 1.22) and decreased frequency of rs130058 minor alleles (1.25 vs 1.75). Concerning other predictors, the prominent SNPs rs6311 and rs6313, which showed complete linkage in our sample, did hardly impact classification results. Among other positive reports, rs6311 was associated with ADHD in a rather large candidate gene study, but overall no consistent associations were reported so far ^35,39^.

However, the literature on in vivo SERT binding is limited. A previous study using single-photon emission computed tomography and the radioligand [^123^I]FP-CIT, binding to dopamine and serotonin transporters, did not demonstrate serotonergic binding differences in 17 ADHD patients compared to HC^40^. These findings were supported by a PET study using the tracer [^11^C]MADAM that reported similar mean binding between eight ADHD patients and HC in several ROI, including prefrontal cortex, thalamus and putamen^41^. However, these results must be interpreted in the light of different radioligands and small sample sizes. The most extensive analysis of SERT binding in ADHD reported so far showed overall decreased BP_ND_ in 25 ADHD patients compared to age and sex matched controls applying the current gold-standard radioligand [^11^C]DASB. Strongest effects were observed in the striatum, insula and anterior cingulate cortex; however, these results did not withstand correction for multiple testing in the post hoc analyses. Interestingly, only interregional molecular correlations of SERT binding between the hippocampus and the precuneus withstood correction thresholds, indicating that an interplay of brain regions may better portray abnormalities of serotonergic transmission in ADHD^16^. These thoughts are in line with moderate to high accuracy despite the lack of single predictor association results in our sample.

There is a particularly abundant literature for ADHD classification models to put these results into perspective with. While advanced statistics and especially machine learning methods have been established in all of neuropsychiatric research in recent years, this particular boom in ADHD is probably owed to the heterogeneous nature and lack of objective biomarkers contrasted by overlapping clinical phenotypes with highly subjective symptoms and frequent comorbidities. Usually, diagnostic models were based on EEG or MRI data and aimed at automated classification of ADHD or MDD and HC.

Thereby, no algorithm proved to be superior to the other frequently applied techniques such as RF, support vector machines (SVM), neural networks or gradient boosting machines. This observation has been shared throughout different application areas of machine learning and culminated in the “no-free-lunch”-theorem, meaning that comparative algorithm performance cannot be generalized and is dependent on structure and context of the data as well as the model^42^. Nevertheless, RF and SVM may be the most commonly applied and best-established algorithms in imaging-based research^10,43^. While SVM was demonstrated to often outperform RF by means of accuracy^44^, RF may be more resilient to overfitting in small datasets as no hyper-parameter tuning is necessary and the generalization error does not increase with trees grown^22^. Furthermore, in contrast to SVM, both feature selection and classification can be performed with RF. Consequently, for this investigation all analyses were performed with RF.

A contemporary review suggested accuracy between 0.6 and 0.8 for published MRI based algorithms that conform with methodological standards concerning validation and feature selection in ADHD research^10^. Surveying the reports for MRI based models for ADHD diagnosis, accuracies above 0.9 attract attention^43^. However, higher accuracies reported by some imaging studies may be owed to circular analysis or other intrusions of information between training and test samples. Although EEG based machine learning algorithms have been supported by the Food and Drug Association (FDA), preliminary results are hindered by the same issues ^45^.

The focus of this study on genetic imaging applying PET instead of in comparison easily obtainable MRI data brings about a considerably extenuated sample size of 34 subjects compared to some reported MRI based classification algorithms. While there is no other PET imaging and genetic machine learning study for comparison, study populations from MRI studies ranged from few dozens to several hundred subjects. Interestingly, recent meta analyses and reviews have emphasized a curious finding of decline of accuracy with increased sample size across studies despite oppositional effects within studies^46,47^. While the majority of published studies featured below 100 observations, a decline on accuracy with sample size was observed. This may partly be explained by the contrast of narrow study settings to the heterogeneity of phenotypes in the clinical routine, which are better reproduced by larger, more natural samples. Along these lines, larger samples are usually collected in multi-center approach and slight differences in implementation of study protocols or data acquisition and interpretation among contributing centers may explain a reduction in accuracy as machine learning algorithms can easily be disrupted by data disparity. On the other hand, however, accuracies may be inflated in small samples despite optimal validation protocols. Furthermore, smaller studies may be more prone to publication bias as low accuracy samples are probably underreported.

Our results must be interpreted cautiously due to the lack of external validation, constituting the most important limitation. The latter was not possible as to our knowledge there was no comparative sample on SERT binding in ADHD measured with [11 C]DASB that could have been used for validation. Thus, we cannot rule out overoptimistic PPV and NPV in our model. Although 38 subjects can be considered a large sample for a PET neuroimaging analysis, the observation count is marginal for machine learning classification. The CV design with fold-specific feature selection, refrainment from further parameter tuning and averaging across repeats can be regarded as state of the art but cannot substitute low sample size and lack of external validation^24^. Along these lines, the moderate standard deviation across the repeats of the CV procedure must be noted and indicates that results may still be dependent on the data context. In line with these considerations, prediction accuracy based on MRI data from a recently published large sample of MDD patients thoroughly analyzed throughout a machine learning competition did not surpass 0.65^43^. These lower accuracies but may be closer to the actual clinical value of currently available models. While an accuracy higher than 0.8 generally indicates good discrimination^48^, the cut-offs necessary for clinical application are primarily dependent on already available screening and diagnostic tests and the expected ratio of observed cases. For example, an easily applicable screening test designed for detecting the few cases among a predominant number of controls must show good sensitivity while diagnostic tests also need high specificity to prevent false-positive outcomes. Considering the cost-intensive nature of PET and, to a lesser degree, also MRI, an imaging-based classification model can currently only fulfill a role as specialized diagnostic tool in clinically challenging cases as proposed for classification of psychosis^49^. Consequently, current data do not support the viability of solely imaging-based algorithms for clinical applications, neither regarding ADHD nor MDD. Along these lines, clinical predictors such as scores of the Conners’ Adult ADHD Rating Scale can most likely increase accuracies but were kept out of this analysis as two clearly distinct samples, healthy controls and ADHD patients, were compared. Keeping in mind the symptom overlap between ADHD and frequent comorbidities, a clinical and bio-marker based transdiagnostic classification model may be clinically meaningful even with moderate accuracy.

To summarize, we propose a diagnostic prediction model for ADHD and HC based on multimodal serotonergic data. Thereby, we present the first PET based classification model for ADHD and expand on previous designs based solely on a single data type. We cannot yet advocate clinical applicability of this diagnostic model but present a step towards the goal of precision medicine in psychiatry. More importantly, our findings support different serotonergic profiles in ADHD and HC, reflected by distinct SERT and *HTR1B* as well as *HTR2A* activity, and especially put emphasis on the rs130058 and rs1328684 polymorphisms.
